# Microsatellite marker-assisted backcross breeding for improvement of wheat salt tolerance using Kharchia 65

**DOI:** 10.1186/s12864-024-10468-y

**Published:** 2024-06-01

**Authors:** Kritika Sharma, Shikha Yashveer, Vikram Singh, Sudhir Sharma, Mandeep Redhu, Mehdi Rahimi

**Affiliations:** 1 Department of Molecular Biology, Biotechnology and Bioinformatics, COBS&H, CCS Haryana Agricultural University, Hisar, India; 2 Department of Genetics and Plant Breeding, COA, CCS Haryana Agricultural University, Hisar, India; 3 Department of Plant, Soil and Agricultural System, College of Agricultural, Life and Physical Sciences, Southern Illinois University, Carbondale, USA; 4 Department of Biotechnology, Institute of Science and High Technology and Environmental Sciences, Graduate University of Advanced Technology, Kerman, Iran

**Keywords:** *Nax*1 and *Nax*2, Salinity, SSR markers, Polymorphism

## Abstract

**Background:**

Salinity is a significant abiotic stress that affects plants from germination through all growth stages. This study was aimed to determine the morpho-physiological and genetic variations in BC_1_F_2_, BC_2_F_1_ and F_3_ generations resulting from the cross combination WH1105 × Kharchia 65.

**Results:**

A significant reduction in germination percentage was observed under salt stress in BC_1_F_2_ and F_3_ seeds. Correlation, heritability in the broad sense, phenotypic coefficient of variability (PCV) and genotypic coefficient of variability (GCV) were measured for all traits. The presence of both *Nax1* and *Nax2* loci was confirmed in twenty-nine plants using the marker-assisted selection technique. Genetic relationships among the populations were assessed using twenty-four polymorphic SSR markers.

**Conclusion:**

Cluster analysis along with two and three-dimensional PCA scaling (Principal Component Analysis) revealed the distinct nature of WH 1105 and Kharchia 65. Six plants closer to the recurrent parent (WH1105) selected through this study can serve as valuable genetic material for salt-tolerant wheat improvement programs.

**Supplementary Information:**

The online version contains supplementary material available at 10.1186/s12864-024-10468-y.

## Background

Bread wheat (*Triticum aestivum* L. em. Thell) is a staple and economically important crop in India. Like many modern crops, wheat production is critically vulnerable to various abiotic stresses such as extreme temperatures, drought, alkalinity, salinity, and pathogen infection. Salinity, among the abiotic stresses, is one of the major factors that adversely affect the germination and growth stages of wheat ultimately reducing yield. Salinity affects wheat plants through various means viz., physiological drought, ion toxicity, nutritional imbalances, oxidative stress, alteration of metabolic processes, membrane disruption, and reduction of cell division and expansion [1–5]. The initial ionic and osmotic stresses inhibit or delay seed germination by preventing water absorption and seed imbibition. Additionally, sodium and chloride ions have toxic effects on germinating seeds [6]. Globally, 831 million hectares of land are affected by salinity, including 397 million hectares of saline soils and 434 million hectares of sodic soils. In India, 6.75 million hectares of land is salt affected [7], a number expected to increase up to 20 million hectares by the end of the 21st century. Given the growing concern over salinity problem in many parts of the world and wheat’s susceptibility to it, developing salinity-tolerant wheat cultivars is crucial for ensuring global food security.

Various approaches have been used to develop wheat genotypes that can thrive under saline conditions and response well to high-input farming. Molecular breeding has been extensively employed for wheat improvement, involving foreground selection to identify plants with desirable genes in segregating populations and background selection, using polymorphic markers to ensure sufficient recovery of the recurrent parent genome (RPG). The *Nax1* and *Nax2* loci contain candidate genes of *TmHKT1*; *4-A2* and *TmHKT1; 5-A* family respectively. These genes are responsible for sodium exclusion and provide salinity tolerance to wheat plants. The *Nax1* locus reduces sodium concentration in leaf blades by retaining sodium in the leaf sheath, while *Nax2* improves discrimination between sodium and potassium at the point of xylem loading and reduces the transport of sodium from root to shoot [8]. The introgression of *Nax1* from durum wheat (*Triticum turgidum* ssp. durum) to bread wheat has been shown to reduce the leaf sodium concentration by 50%, while *Nax2* reduced it by 30% and both genes together reduced it by 60% in wheat plants grown under salt stress [9]. *Nax1* promotes leaf longevity, better photosynthetic rate and stomatal conductance [10]. ‘Kharchia-65’ is a salt-tolerant variety native to the village of ‘Kharchi’ in the ‘Pali’ district of Rajasthan, capable of withstanding up to pH 9.4, making it suitable candidate for developing salt-tolerant varieties worldwide [11, 12]. WH 1105 is a high yielding cultivar from the Northwest Plain Zone of India, resistant to yellow and brown rusts, flag smut, leaf blight and powdery mildew diseases. It exhibits wide adaptability with nutritional qualities such as high protein content (12.4%) and a good *chapatti-*making score (7.60). The salt tolerance of Kharchia-65 and agronomic characteristics of WH1105 make them ideal parents for crossing to obtain potential genotypes with salt tolerance traits. In the current study, a two-tier approach was conducted to develop crosses between high-yielding wheat genotypes WH1105 and Kharchia, followed by a screening of different generations (BC_1_F_2_, BC_2_F_1_ and F_3_) for morpho-physiological variability at the field and molecular levels using marker-assisted selection for *Nax* loci.

## Materials and methods

Plant material was obtained from the Wheat and Barley section, Department of Genetics and Plant Breeding, CCS Haryana Agricultural University, Hisar. The experimental material consisted of three generations including high-yielding wheat genotypes WH1105 and Kharchia and subsequent screening of different generations (BC_1_F_2_, BC_2_F_1_ and F_3_) derived from a cross between WH1105 and Kharchia-65. The experiment was carried out during the Rabi season at the Department of Molecular Biology, Biotechnology and Bioinformatics, CCS Haryana Agricultural University, Hisar. Seeds were sown in trays under chloride-dominated salt stress (ECe ∼ 8 ds/m). Healthy germinated seedlings were transplanted to pots and supplemented with Hoagland’s nutrient solution till maturity. Various traits were recorded for statistical analysis, including germination percentage, plant height (cm), number of tillers per plant, ear length (cm), number of grains per ear, number of spikelets per spike, number of grains per plant, grain yield per plant (g), 1000-grain weight (g), biological yield per plant (g), harvest index (%) and main spike weight (g). Correlation analyses was performed using the Pearson correlation coefficient (r) to determine the relationship among all traits. Genotypic and phenotypic coefficients of variation along with Pearson correlation coefficients (r) and broad sense heritability were calculated.

For the marker-assisted selection, genomic DNA was isolated using the CTAB extraction method [13] from the young leaf samples. Quantification of DNA samples was done by 0.8% agarose gel electrophoresis. The presence of Nax loci was checked using PCR amplification of Nax1 (F 5’-ATCGCATGATGCACGTAGAG-3’) and R 5’-ACATGCATGCCTACCTAATGG-3’) and Nax2 (F 5’-TCTCCATCATTCAACATCAATCG-3’ and R 5’-TGTAGCTCGTCGGGGTGTGTTGC-3’) primers. PCR was performed in 20 µl reactions containing 50–100 ng of DNA template, 20 µl containing 10X PCR buffer, 10 mM dNTPs, 0.4 µM of each primer, 1-unit Taq DNA polymerase using My-CYCLER (programmable thermal cycler from BIORAD™ INTERNATIONAL). Additionally, one hundred SSR markers were screened to check polymorphism between the parents. A dendrogram illustrating the genetic relationship among all plants was generated using the unweighted pair-group arithmetic average (UPGMA) method through NTSYS PC software (Numerical Taxonomy system, Applied Biostatistics, NY) [14]. A principal component analysis (PCA) was also conducted.

## Results and discussion

### Morpho-physiological analysis

Salt stress negatively affected seed germination, seedling emergence and seedling establishment. High concentrations of soluble salts in the soil led to a reduction in germination percentage and speed of seed emergence of many plant species [15]. A significant decrease in germination percentage was observed in BC_1_F_2_ (18%) and F_3_ (40%) seeds under salt stress (ECe ∼ 8ds/m). This reduction was attributed to reduce water absorption from the soil, resulting in increased osmotic pressure due to the high salt content in the soil solution. Similar findings have been reported in previous studies [16, 17].

Salinity significantly affected the morphological traits of wheat in all three generations. Correlation analysis revealed positive association between grain yield and various independent variables, indicating the relative significance of these traits in improving yield. A positive correlation of grain yield per plant was observed at 1% significance with the number of tillers per plant (0.672 and 0.652), biological yield per plant (0.831 and 0.854), 100-grain weight (0.467 and 0.491) and number of grains per plant (0.847 and 0.830) in BC_1_F_2_ and F_3_ generation (Tables 1 and 2).

**Table 1:** Phenotypic correlation coefficients in F_3_ population derived from a cross between WH1105 x Kharchia 65

F_3_	Plant height (cm)	Ear length (cm)	No. of tillers /plant	Number of spikelets/ spike	Main spike weight (g)	Biological yield/plant (g)	Number of grain /ear	Number of grain /plant	Grain yield/plant (g)	100 grain weight (g)	Harvest index main spike (%)	Harvest index (%)
**Plant height (cm)**												
**Ear length (cm)**	0.205^**^											
**No. of tillers /plant**	0.379^**^	0.089										
**Number of spikelets/spike**	0.129^*^	0.278**	0.131*									
**Main spike weight (g)**	0.264^**^	0.123	0.167**	0.147*								
**Biological yield/plant (g)**	0.448^**^	0.074	0.822**	0.139*	0.239**							
**Number of grain /ear**	0.251^**^	0.119	0.219**	0.280**	0.225**	0.315**						
**Number of grain /plant**	0.384^**^	0.044	0.669**	0.145*	0.197**	0.791**	0.506**					
**Grain yield/plant (g)**	0.396^**^	-0.047	0.652**	0.04	0.201**	0.854**	0.360**	0.830**				
**100 grain weight (g)**	0.148^*^	-0.144*	0.260**	-0.104	0.086	0.384**	-0.165**	-0.018	0.491**			
**Harvest index main spike (%)**	0.250^**^	-0.085	0.224**	0.135*	0.279**	0.335**	0.321**	0.332**	0.402**	0.235**		
**Harvest index (%)**	-0.077	-0.150^*^	-0.357^**^	-0.118	-0.016	-0.288^**^	0.178^**^	0.066	0.191^**^	0.138^*^	0.265^**^	

*and ** significant atthe*P* < ***0.05*and the*P*** < ***0.01*levels of probability, respectively.**

**Table 2:** Phenotypic correlation coefficient in BC_2_F_1_ (below diagonal) and BC_1_F_2_ (above diagonal) populations

Traits	Plant height (cm)	Ear length (cm)	No. of tillers /plant	Number of spikelets/ spike	Main spike weight (g)	Biological yield/plant (g)	Number of grain /ear	Number of grain /plant	Grain yield/plant (g)	100 grain weight (g)	Harvest index main spike (%)	Harvest index (%)
**Plant height (cm)**	1	0.113	0.385**	0.06	0.068	0.412**	0.333**	0.309**	0.382**	0.211**	0.261**	-0.03
**Ear length (cm)**	0.395^*^	1	0.065	0.493**	-0.016	0.01	0.017	0.202**	0.001	-0.330**	-0.176*	-0.035
**No. of tillers /plant**	0.198	0.552^**^	1	0.14	0.041	0.727**	0.275**	0.595**	0.672**	0.346**	0.188*	-0.162*
**Number of spikelets/spike**	0.242	0.046	0.085	1	-0.072	0.133	0.088	0.247**	0.118	-0.206**	-0.165*	-0.048
**Main spike weight (g)**	-0.132	-0.16	0.179	-0.197	1	0.073	-0.08	-0.088	-0.055	-0.008	-0.028	-0.219**
**Biological yield/plant (g)**	-0.023	0.105	0.564^**^	0.26	0.142	1	0.383**	0.695**	0.831**	0.433**	0.267**	-0.295**
**Number of grain /ear**	0.182	-0.159	-0.091	0.234	0.01	0.115	1	0.581**	0.553**	0.069	0.385**	0.305**
**Number of grain /plant**	0.047	0.461^*^	0.556^**^	-0.014	0.204	0.483^**^	0.145	1	0.847**	-0.006	0.13	0.205**
**Grain yield/plant (g)**	0.112	0.232	0.560^**^	0.247	0.047	0.756^**^	0.286	0.756^**^	1	0.467**	0.253**	0.233**
**100 grain weight (g)**	0.079	-0.409^*^	-0.318	0.29	-0.201	-0.123	0.108	-0.821^**^	-0.317	1	0.226**	0.052
**Harvest index main spike (%)**	-0.038	-0.03	0.003	0.069	-0.27	0.263	0.335	0.129	0.163	0.016	1	0.057
**Harvest index (%)**	0.197	0.091	-0.201	-0.064	-0.14	-0.535^**^	0.244	0.217	0.134	-0.205	-0.217	1

*and ** significant at*the****P*** < ***0 .05*and the*P*** < ***0.01*levels of probability, respectively.**

Studies on inter-character associations in BC_2_F_1_ generation revealed a significant and positive association of grain yield per plant with the number of tillers per plant (0.560), biological yield per plant (0.756), and number of grains per plant (0.756) (Tables 1 and 2). A similar association of traits with grain yield was reported by [18, 19], indicating that these traits can be further used to select high-yielding plants in wheat improvement programs under salinity.

Estimation of genetic variation along with heritability and genetic advance provide a better idea about the efficiency of selection. A higher phenotypic coefficient of variability (PCV) was recorded than the genotypic coefficient of variability (GCV) for all traits, indicating the influence of the environment (Tables 3 and 4). A perusal of these results revealed maximum phenotypic variations (64.46% and 79.55%) and genotype variations (53.5% and 60.84%) in BC_1_F_2_ and F_3_ generations respectively for the trait grain yield/plant (g). BC_2_F_1_ showed maximum GCV for the number of grains/plant (50.98) and maximum PCV for grain yield per plant (89.33).

**Table 3:** Estimate of genetic and phenotypic parameters for various traits in F_3_ population

Traits	Mean ± SE	Range	GCV (%)	PCV (%)	Heritability (%)	Genetic advance as % of mean 5%
**Plant height (cm)**	75.09 ± 0.68	99.00–38.00	14.36	28.52	25.37	14.91
**Ear length (cm)**	10.27 ± 0.11	16.50–5.40	18.48	29.19	40.06	24.09
**No. of tillers /plant**	2.88 ± 0.07	6.00–1.00	46.41	62.72	54.75	70.74
**Number of spikelets/spike**	16.13 ± 0.13	25.00–11.00	13.23	14.47	83.59	24.92
**Main spike weight (g)**	2.17 ± 0.04	2.98–1.01	60.75	74.32	66.82	102.3
**Biological yield/plant (g)**	8.47 ± 0.28	19.56–1.01	40.6	45.69	78.96	85.71
**Number of grains/ear**	42.84 ± 0.83	72.00–12.00	32.16	37.44	73.77	75.96
**Number of grains/plant**	86.16 ± 2.82	205.00–15.00	50.66	54.86	85.26	112.17
**Grain yield/plant (g)**	2.46 ± 0.09	6.97 − 0.20	60.84	79.55	58.49	27.27
**100 grain weight (g)**	2.87 ± 0.06	4.47–1.03	42.13	47.94	77.23	83.69
**Harvest index main spike (%)**	39.32 ± 0.46	46.97–16.76	28.93	34.02	72.3	69.7
**Harvest index (%)**	30.39 ± 0.56	46.88–10.66	36.99	41.55	79.26	75.38

**Table 4:** Estimate of genetic and phenotypic parameters for various traits in BC_1_F_2_ and BC_2_F_1_ populations

Traits	Populations	Mean ± SE	Range	GCV (%)	PCV (%)	Heritability (%)	Genetic advance as % of mean (5%)
**Plant height (cm)**	**BC**_**1**_ **F**_**2**_	73.14 ± 0.80	102.00-44.50	14.24	25.53	31.11	16.36
	**BC**_**2**_ **F**_**1**_	98.11 **±** 2.15	118.00–76.00	11.6	15.99	52.63	17.34
**Ear length (cm)**	**BC**_**1**_ **F**_**2**_	10.97 ± 0.10	15.50–7.30	12.5	25.27	24.48	12.74
	**BC**_**2**_ **F**_**1**_	12.41 **±** 0.26	15.00-10.50	11.06	25.67	18.57	9.82
**No. of tillers /plant**	**BC**_**1**_ **F**_**2**_	2.58 ± 0.07	5.00–1.00	41.43	53.32	60.39	66.33
	**BC**_**2**_ **F**_**1**_	16.75 **±** 1.21	27.00–6.00	46.97	51.14	84.36	104.64
**Number of spikelets/spike**	**BC**_**1**_ **F**_**2**_	18.16 ± 0.22	29.00–13.00	13.83	16.57	69.67	31.16
	**BC**_**2**_ **F**_**1**_	19.00 **±** 0.31	25.00–17.00	11.6	18.27	40.29	8.33
**Main spike weight (g)**	**BC**_**1**_ **F**_**2**_	1.86 ± 0.031	2.75–1.02	26.26	52.37	25.14	27.12
	**BC**_**2**_ **F**_**1**_	2.91 **±** 0.11	3.92–2.11	21.41	26.91	63.31	35.1
**Biological yield plant**	**BC**_**1**_ **F**_**2**_	9.38 ± 0.344	19.58–1.154	41.55	45.04	85.1	90.77
	**BC**_**2**_ **F**_**1**_	58.64 **±** 3.36	84.00–34.00	38.31	42.34	81.89	87.11
**Number of grains/spike**	**BC**_**1**_ **F**_**2**_	50.97 ± 1.041	74.00–13.00	25.7	30.98	68.85	62.7
	**BC**_**2**_ **F**_**1**_	645.50 **±** 57.06	1355.00-260.00	50.98	54.98	85.97	113.24
**Number of grains/plant**	**BC**_**1**_ **F**_**2**_	91.52 ± 3.372	294.00–13.00	45.15	50.34	80.44	102.92
	**BC**_**2**_ **F**_**1**_	645.50 **±** 57.06	1355.00-260.00	50.98	54.98	85.97	113.24
**Grain yield/plant (g)**	**BC**_**1**_ **F**_**2**_	2.90 ± 0.115	7.03–0.37	53.35	64.46	68.48	25
	**BC**_**2**_ **F**_**1**_	20.75 **±** 1.08	29.47–11.45	48.27	89.33	29.2	53.74
**100 grain weight (g)**	**BC**_**1**_ **F**_**2**_	3.18 ± 0.062	4.47–0.61	29.07	34.34	71.64	50.68
	**BC**_**2**_ **F**_**1**_	3.51 **±** 0.15	4.43–1.73	25.53	65.1	15.38	20.63
**Harvest index main spike (%)**	**BC**_**1**_ **F**_**2**_	40.94 ± 0.386	47.79–22.26	15.34	17.47	77.09	35.45
	**BC**_**2**_ **F**_**1**_	41.99 **±** 0.59	46.67–32.90	23.3	29.32	63.14	60.31
**Harvest index (%)**	**BC**_**1**_ **F**_**2**_	32.07 ± 0.645	46.56–15.27	22.56	26.67	71.54	50.45
	**BC**_**2**_ **F**_**1**_	36.50 **±** 1.34	46.64–18.92	25.2	30.05	70.3	54.5

In BC_1_F_2_, high heritability was observed in biological yield/plant (85.10) and high genetic advance for a number of grains/plant (102.92). High heritability coupled with high genetic advance as a percent of the mean was recorded for the number of grains/plant in F_3_ and BC_2_F_1_ generations. Estimation of variability revealed a higher PCV value than the respective GCV value for all the considered traits indicating minimum influence of environment. These results are in line with [20–24] indicating the presence of environmental influence to some degree in the phenotypic expression of the characters. Grain yield/ plant showed maximum PCV 64.46% (BC_1_F_2_), 79.55% (F_3_) and 89.33% (BC_2_F_1_), similar to what was reported by [25, 26]. High estimates of heritability associated with high estimates of genetic advance were observed for days to 50% flowering and plant height, demonstrating the presence of additive gene effects and indicating the effectiveness of selection for the improvement of these traits. These results aligned with the findings of [27].

### Screening of wheat genotypes for *Nax1* and *Nax2* loci

Selection based on the phenotypic data is not efficient enough as morphological traits are influenced by environmental conditions. Advancement in biotechnology-generated molecular markers such as RFLPs AFLP, RAPD, SSRs and SNPs plays a vital role in the selection procedure. Molecular markers associated with the target gene increase the efficiency of recovering desired traits in the population. SSR markers have been extensively used for tagging and mapping various important genes/QTLs for biotic and abiotic stress in wheat. *Nax*1 and *Nax*2 (SSR) markers associated with salinity tolerance were reported in durum wheat [28–31]. Genomic DNA was isolated from young leaf tissues of the parents and 101 BC_1_F_2_, 53 F_3_ and 30 BC_2_F_1_ plants. The presence of *Nax1* and *Nax2* loci was tested in segregating populations to screen for salt sensitivity. PCR amplification confirmed the presence of *Nax1* and *Nax2* loci in salt tolerant (Kharchia 65) but absent in salt susceptible (WH1105) with band sizes of approximately 225 and 250 bp respectively. A total of ninety-eight plants comprising 57 BC_1_F_2_, 17 F_3_ and 24 BC_2_F_1_ confirmed the presence of the *Nax*1 locus. The presence of the *Nax*2 locus was confirmed in forty-nine plants comprising 32 BC_1_F_2_, 9 F_3_ and 8 BC_2_F_1_. Both *Nax1* and *Nax2 loci* were present in twenty-nine plants (16 BC_1_F_2_, 6 F_3_ and 7 BC_2_F_1_).

### Microsatellite analysis

Screening of segregating populations with polymorphic markers revealed the similarity of progenies with parents. Polymorphism between parents was screened with one hundred fifty-one SSR primer pairs covering almost all the chromosomes of wheat. Twenty-four SSR primer pairs were found to be polymorphic between Kharchia 65 and WH1105 and further used to genotype the populations (Supplementary Table 1). Data counting and scoring were done using a binary number system for the presence and absence of bands as 1 and 0 respectively. Scoring data obtained using SSR primers were further analyzed using the ‘SIMQUAL” sub-program of software NTSYS-pc. Dendrogram, two and three-dimensional PCA scaling representing the genetic relationship among all plants revealed that two parental genotypes (WH1105 and Kharchia 65) were quite distinct. BC_1_F_2_, BC_2_F_1_ and F_3_ plants were interspersed between the two parental lines (Fig. S1-9). Six plants were selected based on the maximum similarity coefficient with the recurrent parent which can be used further for salinity improvement programs. These results conformed with [32, 33] in marker-assisted backcross breeding for rust resistance in wheat. The current research characterized the plants at a molecular level and found significant phenotypic and genotypic variations in all three generations. Screening of segregating populations with polymorphic markers revealed the similarity of progenies with parents. Plants closer to the recurrent parent were selected based on the dendrogram and two-dimensional and three-dimensional PCA scaling. The NTSYS-PC Dendrogram, two and three-dimensional PCA scaling revealed the relationship between progenies and parents.

## Conclusion

The results from the current study highlight the potential use of an old salt-tolerant cultivar (Kharchia-65) for improving the salinity tolerance of a conventionally well-suited variety (WH 1105) by utilizing a marker-based selection approach. The current study reports the successful selection of twenty-nine lines consisting of both Nax1 and Nax2 loci using 24 polymorphic SSR markers for a faster selection of parental (WH 1105) genetic background. Genetic material from this study will potentially lead to the development of a high-yielding variety (like WH 1105) with significant salt tolerance capabilities (like Kharchia-65) that can be continued for production in the increasingly salinized soils around the Northwestern plains of India.

### Electronic supplementary material

Below is the link to the electronic supplementary material.

Supplementary Material 1

Supplementary Material 2

## Data Availability

Data are contained within the article.
