# Supplementary material for: Microsatellite marker-assisted backcross breeding for improvement of wheat salt tolerance using Kharchia 65
Source: BMC Genomics. 2024 Jun 1;25:550. doi: 10.1186/s12864-024-10468-y (PMC11144334; doi:10.1186/s12864-024-10468-y)
Supplement: Supplementary file 1 — Supplementary Material 1 [file 12864_2024_10468_MOESM1_ESM.docx]

**Figure S1: Dendrogram showing relationship between plants of BC1F2 population of WH1105 x Kharchia65 along with both parents generated by UPGMA analysis using polymorphic SSR primers**

**Figure S2: Two dimensional PCA scaling of WH 1105 x Kharchia 65 derived BC_1_F_2_ plants and parental wheat genotypes**

**Figure S3: Three dimensional PCA scaling of WH 1105 x Kharchia 65 derived BC_1_F_2_ plants and parental wheat genotypes**

**Figure S4: Dendrogram showing relationship among WH 1105 x Kharchia 65 derived F_3_ plants along with both parents generated by using 24 polymorphic SSR primers.**

**Figure S5: Two dimensional PCA scaling of WH 1105 x Kharchia 65 derived F_3_ plants along with parental wheat genotypes**

**Figure S6: Three dimensional PCA scaling of WH 1105 x Kharchia 65 derived F_3_ plants and parental wheat genotypes**

**Figure S7: Dendrogram showing relationship among WH 1105 x Kharchia 65 derived BC_2_F_1_ plants along with both parents generated by using 24 polymorphic SSR primers**

**Figure S8: Two dimensional PCA scaling of WH 1105 x Kharchia 65 derived BC_2_F_1_ plants along with parental wheat genotypes.**

**Figure S9: Three dimensional PCA scaling of WH 1105 x Kharchia 65 derived BC_2_F_1_ plants and parental wheat genotypes**
