# Supplementary material for: Microsatellite marker-assisted backcross breeding for improvement of wheat salt tolerance using Kharchia 65
Source: BMC Genomics. 2024 Jun 1;25:550. doi: 10.1186/s12864-024-10468-y (PMC11144334; doi:10.1186/s12864-024-10468-y)
Supplement: Supplementary file 2 — Supplementary Material 2 [file 12864_2024_10468_MOESM2_ESM.docx]

**Supplementary Table 1: List of 24 SSR primers showing polymorphism between parental genotypes (Kharchia 65 and WH1105)**

| **Sr. No.** | **Locus** | **Forward Primer (5' to 3')** | **Reverse Primer (5' to 3')** |
| --- | --- | --- | --- |
| **1** | **Xbarc 10 -2B** | GCGTGCCACTGTAACCTTTAGAAGA | GCGAGTTGGAATTATTTGAATTAAACAAG |
| **2** | **Xbarc159-2B** | CGCAATTTATTATCGGTTTTAGGAA | CGCCCGATAGTTTTTCTAATTTCTGA |
| **3** | **Xbarc240-5B** | AGAGGACGCTGAGAACTTTAGAGAA | GCGATCTTTGTAATGCATGGTGAAC |
| **4** | **Xbarc98-2B** | CCGTCCTATTCGCAAACCAGATT | GCGGATATGTTCTCTAACTCAAGCAATG |
| **5** | **Xcfa2121-5A** | TAAATGGCCATCAAGCAATG | GCTTGTGAACTAATGCCTCCC |
| **6** | **Xcfd18-5D** | CATCCAACAGCACCAAGAGA | GCTACTACTATTTCATTGCGACCA |
| **7** | **Xgwm 601-4A** | ATCGAGGACGACATGAAGGT | TTAAGTTGCTGCCAATGTTCC |
| **8** | **Xgwm533-3B** | AAGGCGAATCAAACGGAATA | GTTGCTTTAGGGGAAAAGCC |
| **9** | **Xgwm537-7B** | ACATAATGCTTCCTGTGCACC | GCCACTTTTGTGTCGTTCCT |
| **10** | **Xgwm 261** | CTCCCTGTACGCCTAAGGC | CTCGCGCTACTAGCCATTG |
| **11** | **Xgwm 356-2A** | AGCGTTCTTGGGAATTAGAG | CCAATCAGCCTGCAACAAC |
| **12** | **Xgwm46-7B** | GCACGTGAATGGATTGGAC | TGACCCAATAGTGGTGGTCA |
| **13** | **Xgwm 601-4A** | ATCGAGGACGACATGAAGGT | TTAAGTTGCTGCCAATGTTCC |
| **14** | **Wmc382-2A** | CATGAATGGAGGCACTGAAA | CCTTCCGGTCGACGCAAC |
| **15** | **Wmc11-3A** | TTGTGATCCTGGTTGTGTTG | CACCCAGCCGTTATATATG |
| **16** | **Xgwm533-3B** | AAGGCGAATCAAACGGAATA | GTTGCTTTAGGGGAAAAGCC |
| **17** | **Xgwm537-7B** | ACATAATGCTTCCTGTGCACC | GCCACTTTTGTGTCGTTCCT |
| **18** | **Xbarc 12-3A** | CGACAGAGTGATCACCCAAATATAA | CATCGGTCTAATTGTCAATGTA |
| **19** | **Barc 065-1B** | CCCATGGCCAAGTATAATAT | GCGAAAAGTCCATAGTCCATAGTCTC |
| **20** | **Xbarc 181-1B** | CGCTGGAGGGGGTAAGTCATCAC | GCGCAAATCAAGAACACGGGAGAAAGAA |
| **21** | **Xwmc 44-1B** | GGTCTTCTGGGCTTTGATCCTG | TGTTCCTAGGGACCCGTAGTGG |
| **22** | **Wmc 364-7B** | ATCACAATGCTGGCCCTAAAAC | CAGTGCCAAAATGTCGAAAGTC |
| **23** | **Gwm268** | AGGGGATATGTTGTCACTCCA | TTATGTGATTGCGTACGTACCC |
| **24** | **Xbarc183-2B** | CCCGGGACCACCAGTAAGT | GGATGGGGAATTGGAGATACAGAG |
